# Cesarean section scar ectopic pregnancy - a management conundrum: a case report

**DOI:** 10.1186/s13256-019-2069-9

**Published:** 2019-05-10

**Authors:** Rumbidzai Majangara, Mugove Gerald Madziyire, Cladious Verenga, Marshall Manase

**Affiliations:** Department of Obstetrics and Gynaecology, University of Zimbabwe College of Health Sciences, PO Box A178, Avondale, Harare Zimbabwe

**Keywords:** Cesarean scar ectopic pregnancy, Diagnostic challenge, Management

## Abstract

**Background:**

Cesarean section scar ectopic pregnancies are a rare complication of pregnancy that may follow previous hysterotomy for any cause, uterine manipulation, and *in vitro* fertilization. It has become more common with the increasing number of cesarean sections worldwide. Fortunately, the use of first-trimester ultrasound imaging has led to a significant number of these pregnancies being diagnosed and managed early.

**Case presentation:**

We report a case of a 36-year-old black African patient who had two previous cesarean sections and one previous surgical evacuation. She presented with a type 2 cesarean section scar ectopic pregnancy that was suspected on the basis of transvaginal ultrasound imaging, but not at laparoscopy/hysteroscopy. A bladder adherent to the upper segment of the anterior uterine wall obscured the gestational mass at laparoscopy. There were extensive intracavitary adhesions that interfered with hysteroscopic visualization. This resulted in the original operative procedure being postponed until magnetic resonance imaging confirmed the ectopic location of the pregnancy. The ectopic gestation was subsequently excised, and the uterus was repaired via laparotomy.

**Conclusions:**

It is important for clinicians and radiologists managing women with risk factors for a scar ectopic pregnancy to maintain a high index of suspicion during follow-up. Failure to diagnose and initiate prompt management may lead to uterine rupture, massive hemorrhage, and maternal death.

## Background

Cesarean section scar ectopic pregnancy is a rare complication of pregnancy, occurring in approximately 1 in 2000 pregnancies [1, 2]. Its incidence is rising in parallel with the increase in primary and repeat cesarean sections. Globally, the incidence of primary cesarean section averages 18.6% of all births [3]. A hysterotomy scar ectopic pregnancy has also been reported following myomectomy, uterine evacuation, previous abnormally adherent placentation, manual removal of placenta, metroplasty, hysteroscopy, and *in vitro* fertilization [4].

There are two recognized types of hysterotomy scar ectopic pregnancies. Type 1 develops in the myometrium and grows toward the uterine cavity, whereas type 2 progresses exophytically toward the uterine serosa [4]. Type 2 pregnancies have an ominous prognosis because they may result in spontaneous uterine rupture, hemorrhage, and maternal death. There is potential for loss of fertility should massive hemorrhage necessitate a hysterectomy.

Symptoms include pelvic pain and vaginal bleeding in the first trimester. Many women are asymptomatic at diagnosis. The investigation of choice is transvaginal ultrasound (TVUS), which may be combined with a transabdominal scan for a panoramic view. In equivocal cases, magnetic resonance imaging (MRI) will confirm or refute the diagnosis [1].

Treatment modalities are dependent on the case presentation. Women have been managed expectantly, medically with methotrexate, or surgically [2, 5]. Apart from surgical excision at hysteroscopy or laparoscopy or laparotomy, vacuum aspiration can be used to remove the ectopic scar [6]. This case report aims to expose a diagnostic conundrum that clinicians might face. Cesarean scar ectopic pregnancies are a rare presentation that may be difficult to diagnose and for which a management option may be hard to choose.

## Case report

We describe a case of a 36-year-old black African woman with two previous live births by cesarean section and two previous miscarriages who was referred in her fifth pregnancy after 6 weeks of amenorrhea. Her serum quantitative β-human chorionic gonadotropin (bHCG) was 16,124 mIU/ml. However, an intrauterine or extrauterine pregnancy could not be located on a transabdominal ultrasound scan.

A copper intrauterine contraceptive device had been removed 2 months prior to her last menstrual period. She reported using one cycle of clomiphene 50 mg with the hope of achieving a twin pregnancy.

She had delivered twice by cesarean section for failure to progress. Her last two pregnancies had been first-trimester miscarriages; one was managed expectantly, and the other was surgically evacuated, though the actual procedure was unknown to the patient. She did not have any chronic medical illness and was not receiving any medication prior to this presentation. She stayed in a city suburb that was well serviced. She was a school principal in her second marriage with no children in the current relationship. She did not smoke or drink alcohol.

On examination, she had a normal blood pressure of 113/70 mmHg and a pulse rate of 98 beats/min. Her body temperature was 37.5 °C. On examination, her cardiorespiratory and neurological systems were normal. Her abdomen was soft and not tender. The result of her pelvic examination was normal. TVUS showed a gestational sac of 13 mm with irregular margins and a visible yolk sac located on the anterior isthmic portion of the uterus, raising suspicion of a cesarean section scar ectopic pregnancy. She declined a Doppler ultrasound evaluation scheduled for the next day. She was scheduled to have serial bHCG evaluations every 48 h.

**Fig. 1:**
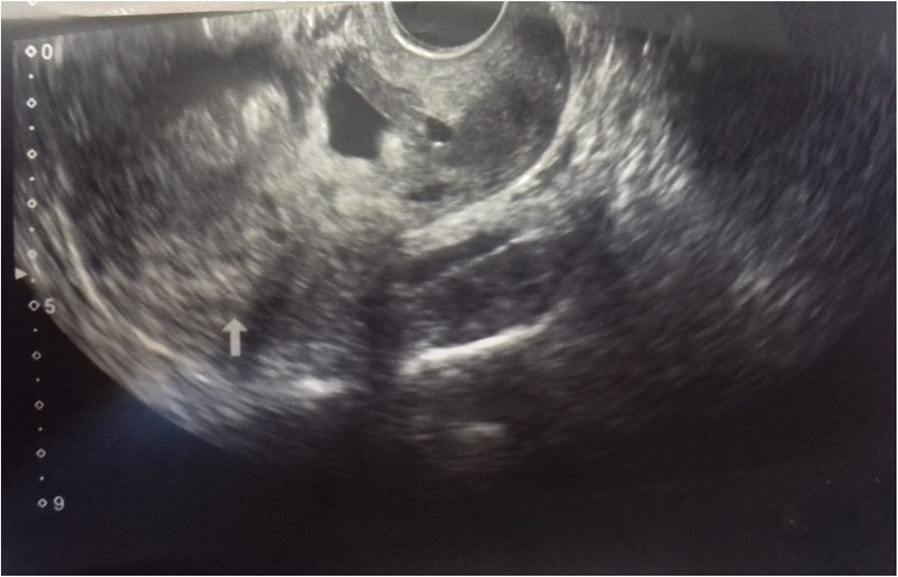
Transvaginal ultrasound scan longitudinal view

A repeat serum quantitative bHCG done 48 h after the initial test revealed a level of 21,521 mIU/ml, a 33% rise. She defaulted follow-up until 1 week later, when she presented with pelvic pain of increasing intensity for 5 days. An urgent transvaginal scan was performed. A fetal pole with active cardiac activity (crown-rump length 0.9 cm) in a gestational sac was located in the anterior low myometrium. The sac traversed the full width of the anterior myometrium, with the posterior margin of the sac abutting the anterior margin of endometrium and the anterior margin of the sac extending to a subserosal location in a fairly exophytic fashion. There was evidence of trophoblastic circulation on Doppler examination. There was no endometrial fluid or free pelvic fluid (Fig. 1).

**Fig. 2:**
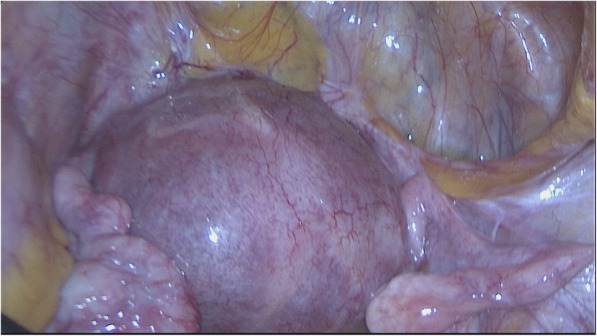
Laparoscopic view. Bladder adherent high on anterior myometrium. Ectopic gestation not visible

**Fig. 3:**
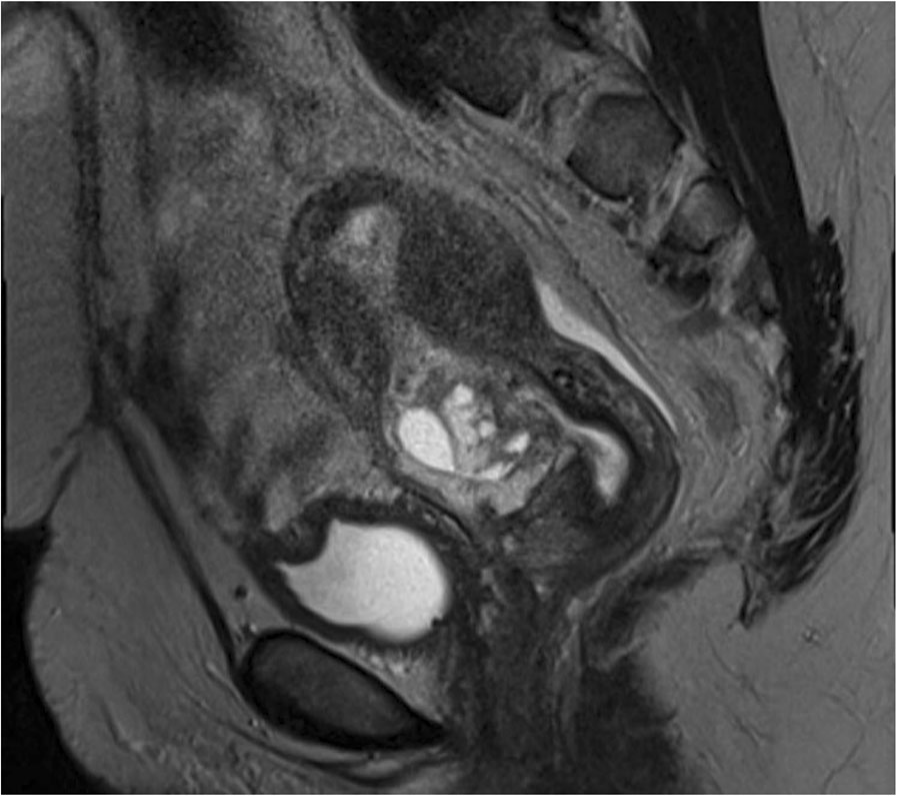
Magnetic resonance imaging of the pelvis, sagittal plane. Ectopic gestation can be seen within the anterior myometrial defect

She was immediately admitted for a diagnostic laparoscopy/hysteroscopy and possible excision of the scar pregnancy if confirmed. A preoperative complete blood count showed hemoglobin 13.4 g/dl, white blood cells 7.2 × 10^3^/μl, and platelet count 243 × 10^3^/μl. The patient’s kidney function was normal with sodium 135 mmol/L, potassium 4.9 mmol/L, urea 3.5 mmol/L, and creatinine 65 μmol/L. The patient’s liver function test results were also normal. She had a negative result in a blood test for human immunodeficiency virus. Urinalysis did not show abnormalities. The patient’s random blood sugar was 5.6 mmol at admission. At laparoscopy, the bladder was adherent high on the anterior uterine wall, and the ectopic pregnancy was not visualized (Fig. 2). At hysteroscopy, there were extensive adhesions within the lower endometrial cavity, which obscured visibility. There was no active intracavitary bleeding ruling out a threatened or inevitable miscarriage. We could not visualize any obvious bulge in the cervical canal suggestive of a cervical ectopic pregnancy. Because of the uncertainty of the location of the pregnancy due to adhesions, excision was postponed. Postoperatively, the patient became unstable with low blood pressure, systolic pressure range of 82 to 95 mmHg and diastolic pressure range of 40 to 55 mmHg, and a pulse rate range of 64 to 73 beats/min, but without active vaginal bleeding or use of medications inducing persistent hypotension. Anesthesia had been induced with etomidate 16 mg and suxamethonium 100 mg, and maintenance was initiated with isoflurane 0.8–1.5%. Intra- and perioperative analgesia was induced with fentanyl 200 mg intravenously (IV), indomethacin 100 mg rectally, and paracetamol 1 g IV. Antibiotic prophylaxis was with ceftriaxone 1 g IV and metronidazole 500 mg IV. This prompted us to order an urgent MRI scan to map the location of the pregnancy in the immediate postoperative period. MRI confirmed the TVUS findings of a cesarean section ectopic scar extending to the serosa (Fig. 3).

An emergency laparotomy was then performed on the same day. The abdomen was entered through a Pfannenstiel incision along the old skin scar. A transverse incision was made in the upper uterine segment just above the adherent bladder. The products of conception were removed with forceps, and the gap in the anterior myometrium at the old scar was seen and felt. There was massive bleeding from the implantation site. Twenty milliliters of vasopressin (20 U diluted in 100 ml) in normal saline was administered into the bleeding myometrium edges. The edges were apposed in layers with VICRYL suture (Ethicon, Somerville, NJ, USA) to repair the defect. Estimated blood loss was 2000 ml. The patient was transfused with 1 U of packed cells intraoperatively. She was continued on the same intravenous antibiotics and analgesia that had been commenced after the laparoscopy. Her hemoglobin count on day 1 postoperatively was 8 g/dl, and she declined any further transfusion. Oral iron and folic acid supplementation was commenced.

The patient’s postoperative recovery was uneventful, and she was discharged on day 4 after surgery. Histology confirmed the presence of decidua and chorionic villi. The patient wanted a child because she was in a new relationship, but she was no longer sure of her future fertility plans after the ectopic pregnancy. A levonorgestrel implant was inserted 2 weeks postoperatively. The patient last attended physical review at 6 weeks, and she was well with no problems related to the surgery at a telephone review at 3 months postoperatively.

## Discussion

We present a case of a 36-year-old patient who had two previous cesarean sections and one previous surgical evacuation of the uterus. She presented initially with a pregnancy of unknown location, then suspected scar ectopic pregnancy followed by an inconclusive laparoscopy and hysteroscopy. She was then managed definitively by excision and repair of the myometrial defect following affirmation of uterine ectopic scar by MRI.

Though our patient had undergone three previous uterine procedures, one case series shows that most scar ectopic pregnancies occurred after only one cesarean section. Hence, the number of cesarean sections appear to have no impact as an independent risk factor [2, 7].

It is known that pregnancy in the presence of an intrauterine contraceptive device is a high risk factor for ectopic pregnancies. There is no information on whether previous use of an intrauterine device is associated with a scar ectopic pregnancy.

As in our patient’s case, uterine scar ectopic pregnancies can pose a diagnostic conundrum. Our initial suspicion was raised at the time of TVUS. The differential diagnosis included cervical ectopic pregnancy, cervicoisthmic pregnancy, and inevitable miscarriage. Known diagnostic criteria for a cesarean section scar pregnancy are a gestational sac located anteriorly at the level of the internal os within a visible myometrial defect and functional trophoblast demonstrated on color Doppler imaging studies [8]. The pelvic adhesions and intrauterine adhesions seen at laparoscopy/hysteroscopy made us lose confidence in the TVUS findings, until MRI affirmation.

Surgical excision was our treatment of choice because the pregnancy was extending exogenously, the fetus had active cardiac activity, and the bHCG levels at diagnosis were very high. Methotrexate has traditionally been reserved for the management of ectopic pregnancies with a bHCG value less than 5000 mIU/ml. Uterine artery embolization and expectant management are options for stable patients but require close follow-up to avoid potential disastrous consequences such as uterine rupture. Surgical treatment, successful in 96%, is the most definitive treatment option that removes the gestation and offers an opportunity to repair the uterine defect and a chance at future fertility [1, 5, 9]. Surgical excision can be achieved via laparotomy, laparoscopy, and hysteroscopy or vacuum aspiration [6], depending on the location of the gestation and the surgeon’s expertise, among other factors. We chose laparotomy because it would give us better access and control of hemorrhage in this situation of type 2 scar ectopic pregnancy. Despite a live birth rate of 57% in one systematic review, 63% of women managed expectantly required hysterectomy for the management of life-threatening hemorrhage following spontaneous uterine rupture or abnormally adherent placenta [6]. The high morbidity and risk of death do not justify expectant management of a viable scar pregnancy.

The risk of recurrent scar ectopic pregnancy is low, 3.2–5.0% [2, 10]. Women who intend to continue childbearing should be informed of the low risk of recurrence but the potential serious sequelae of a recurrence. Even with an intrauterine pregnancy, the woman is still at risk of complications of multiple hysterotomies, such as abnormally adherent placenta, uterine rupture, massive hemorrhage, and hysterectomy. Future pregnancies require meticulous specialist follow-up.

## Conclusions

Uterine scar ectopic pregnancies pose a diagnostic challenge that calls for clinicians and radiologists managing women with associated risk factors to maintain a high index of suspicion during imaging and follow-up. A missed diagnosis with delayed management may lead to uterine rupture, massive hemorrhage, and maternal death. Transvaginal scanning equipment and training should be readily available even in resource-limited settings. A screening tool for evaluating at-risk patients and a protocol for escalating to MRI for equivocal cases should be available at the point of care.

## Abbreviations

bHCG: β-Human chorionic gonadotropin
MRI: Magnetic resonance imaging
TVUS: Transvaginal ultrasound scan
